# Thrombin dynamics and clinical events in cirrhosis: low prothrombin conversion predicts bleeding risk

**DOI:** 10.3389/fcell.2026.1764080

**Published:** 2026-09-01

**Authors:** Ruth Anne Laura Willems, Alberto Zanetto, Elena Campello, Joke Konings, Cristiana Bulato, Matthijs Kramer, Francesco Paolo Russo, Mark Roest, Marco Senzolo, Patrizia Burra, Hugo ten Cate, Judith de Vos-Geelen, Bas de Laat, Paolo Simioni, Romy de Laat-Kremers

**Affiliations:** 1 Department of Functional Coagulation, Synapse Research Institute, Maastricht, Netherlands; 2 Department of Internal Medicine, Maastricht University Medical Center, Maastricht, Netherlands; 3 Thrombosis Expert Center Maastricht, Maastricht University Medical Center, Maastricht, Netherlands; 4 Cardiovascular Research Institute, Maastricht (CARIM), School for Cardiovascular Diseases, Maastricht, Netherlands; 5 Department of Surgery, Oncology and Gastroenterology, University of Padova, Padova, Italy; 6 Gastroenterology and Multivisceral Transplant Unit, Padova University Hospital, Padova, Italy; 7 Department of Medicine (DIMED), University of Padova, Padova, Italy; 8 Internal Medicine and Thrombotic and Haemorrhagic Disease Unit, Padova University Hospital, Padova, Italy; 9 Department of Gastroenterology and Hepatology, Maastricht University Medical Center, Maastricht, Netherlands; 10 GROW—School for Oncology and Reproduction, Maastricht University, Maastricht, Netherlands; 11 Department of Platelet Pathophysiology, Synapse Research Institute, Maastricht, Netherlands; 12 Center of Thrombosis and Haemostasis, Gutenberg University Medical Center, Mainz, Germany; 13 GROW, Research Institute for Oncology and Reproduction, Maastricht University Medical Center, Maastricht, Netherlands; 14 Department of Data Analysis and Artificial Intelligence, Synapse Research Institute, Maastricht, Netherlands; 15 First Chair of Internal Medicine and Thrombotic and Haemorrhagic Disease Unit, Padova University Hospital, Padova, Italy

**Keywords:** bleeding, cirrhosis, prothrombin conversion, thrombin generation, thrombosis

## Abstract

Pro- and anti-coagulant factors are significantly altered in liver cirrhosis resulting in an increased bleeding and thrombosis risk. The thrombin generation (TG) test often seems unaltered in cirrhosis, indicating that a new hemostatic balance is reached. TG is determined by prothrombin conversion and thrombin inactivation. These processes can be quantified using the computational thrombin dynamics (TD) add-on analysis for the TG test. We studied TD parameters in cirrhosis to explore its predictive potential for bleeding and thrombosis. In the current study, 32 CP-A, 22 CP-B and 41 CP-C cirrhosis patients and 20 healthy controls were enrolled. Cirrhosis was predominantly alcohol-related (52%), while viral and metabolic etiologies accounted for 34% and 14% of cases, respectively. Patients receiving anticoagulant therapy were excluded. TG was measured using PPP Reagent Low in the presence and absence of thrombomodulin. TG peak height and velocity index were higher in patients than controls (+20%,p = 0.039 and +70%,p = 0.002), and time-to-peak was shorter (−18%,p < 0.0001). TD parameters were obtained computationally based on TG and coagulation factor data. The amount of prothrombin converted (PC_tot_) was significantly reduced in patients (−43%,p < 0.0001), and the magnitude of the reduction was associated with disease severity. The maximum rate of prothrombin conversion (PC_max_) showed a trend towards higher levels in CP-A patients. On the anticoagulant slide, the thrombin decay capacity (TDC) was significantly lower in patients (−44%,p < 0.0001), indicating a newly developed balance between pro- and anticoagulant processes that result in a slightly procoagulant TG phenotype. Four patients experienced bleeding and seven experienced thrombosis during follow-up (median = 499 days; CI: 478–524 days). Low maximum prothrombin conversion rates (PC_max_) were associated with an increased hazard of bleeding. Patients in the lowest 20% of PC_max_ had a HR of 12.9 (95% CI 1.34–124.31, p = 0.027) for bleeding. Low thrombin-α_2_Macroglobulin (T-α_2_M) complex formation was significantly associated with thrombosis during follow-up. Patients in the lowest quintile of T-α_2_M had a HR of 6.6 (95% CI 1.47–29.9, p = 0.014) for thrombosis. We conclude that reduced prothrombin conversion and thrombin inactivation result in rebalanced TG in cirrhosis patients. Our results demonstrate the potential clinical usefulness of TD parameters for the stratification of bleeding and thrombotic risk in cirrhosis patients.

## Introduction

Cirrhosis induces complex alterations in the hemostatic system due to loss of liver function, which leads to reduced coagulation factor production (Lisman and Leebeek, 2007; Lisman and Porte, 2010; Lisman et al., 2002; Rautou et al., 2023).^.^As a consequence, plasma levels of procoagulant factors FII, FV, FVII, FIX, FX, and FXI, and the anticoagulant factors protein C, protein S, and antithrombin are reduced (Senzolo et al., 2012; Potze et al., 2013; Delahousse et al., 2010; Tripodi et al., 2013). FVIII levels are normal or increased in cirrhosis patients, as extrahepatic FVIII production by the spleen and kidneys increases in patients with liver failure (Lisman and Leebeek, 2007). Besides changes in the coagulation system, thrombocytopenia is a common complication of cirrhosis, which can affect the treatment and surgical procedures due to an increased bleeding risk.

Despite reduced concentrations of pro- and anticoagulant factors, no clear bleeding or thrombotic phenotype exists (Senzolo et al., 2012; Potze et al., 2013; Delahousse et al., 2010; Tripodi et al., 2013). Typically, cirrhosis patients are at risk for both bleeding and thrombosis​ (Lisman et al., 2022)​. Bleeding episodes often present as bruising, bleeding after invasive procedure, or bleeding from ruptured esophageal varices. Bleeding is common in cirrhosis patients, although most bleeding complications do not seem to be directly related to the hemostatic alterations, and are often caused by procedures and portal hypertension ​ (Lisman and Leebeek, 2007; Intagliata et al., 2023).

Portal hypertension leads to increased splanchnic blood pressure and the formation of collateral veins, increasing the risk for bleeding complications (Ferro et al., 2012) (Boks et al., 1986), whereas provoked bleeding complications occur during or after procedures as a result of mechanical injury to vessels (Lisman et al., 2022). Moreover, thrombosis is relatively common in cirrhosis patients and typically presents as either venous thromboembolism (VTE) or portal vein thrombosis (PVT) (Lisman and Porte, 2010; Northup et al., 2006; Sharara and Rockey, 2001; Northup et al., 2008; Mor et al., 1993; Søgaard et al., 2009; Ambrosino et al., 2017; Tripodi et al., 2011a; McMurry et al., 2021; Subhani et al., 2022). In contrast to VTE, for which hypercoagulability is a known risk factor, portal vein thrombosis in cirrhosis patients could not be predicted by the existing laboratory test for the detection of hypercoagulability (Scheiner et al., 2020; T et al., 2021; Prakash et al., 2023). The cirrhosis-associated thrombotic tendency could be caused by the simultaneous decrease in anticoagulant factors, alongside the reduction of procoagulant factors, in combination with an increase of Factor (F) VIII levels. It has been reported previously that coagulation assays measuring procoagulant factors only, such as the prothrombin time, would indicate a bleeding tendency in cirrhosis (Lisman and Leebeek, 2007; Potze et al., 2013; Tripodi et al., 2005; Tripodi et al., 2007). In contrast, global coagulation assays, such as the thrombin generation test, indicate that a new, but precarious hemostatic balance is reached, which is reflected by a normal thrombin generating capacity (Senzolo et al., 2012; Delahousse et al., 2010; Tripodi et al., 2013; Tripodi et al., 2005; Tripodi et al., 2011b; Kleinegris et al., 2014; Lisman et al., 2010; Kremers et al., 2015a; Campello et al., 2021; Lebreton et al., 2020; Sinegre et al., 2023; Singh et al., 2022).

Thrombin generation in plasma is dependent on two underlying processes, the production of thrombin (i.e., prothrombin conversion) and the removal of thrombin from the clotting plasma (thrombin inactivation) (Hemker et al., 2002). The thrombin dynamics method was designed to quantify pro- and anticoagulant processes during TG, by quantifying the rate of prothrombin conversion, i.e., the production of new thrombin, and the subsequent inhibition of thrombin by its natural inhibitors (Kremers et al., 2015a; Kremers et al., 2015b; Kremers et al., 2016). We have previously shown that prothrombin conversion is significantly reduced in liver cirrhosis patients, although the maximal velocity of prothrombin conversion is elevated in most patients (Kremers et al., 2015a). In addition, the reduction of antithrombin levels was reflected in a reduction of the thrombin decay capacity, resulting in a rebalanced thrombin generation profile (Kremers et al., 2017).

In this study, we investigated changes in prothrombin conversion and thrombin inactivation in Child-Pugh (CP) A, B and C cirrhosis patients, especially in association with the individual thrombotic and bleeding tendency of the patients. To investigate the latter, we prospectively explored the potential of thrombin dynamics parameters as a predictor for bleeding and/or thrombotic episodes.

## Methods

### Patient enrollment

As previously described, patients with cirrhosis were prospectively enrolled between 1 June 2022, and 1 May 2023, in the Gastroenterology and Multivisceral Transplant Service of Padova University Hospital (Zanetto et al., 2024). Cirrhosis was diagnosed based on clinical, biochemical, and/or histological manifestations. The Child-Pugh Score was used to estimate disease severity. The relatively high proportion of patients with Child-Pugh C cirrhosis reflects the clinical setting and recruitment strategy of the study, as participants were enrolled at a tertiary referral center specializing in advanced liver disease and liver transplantation. This referral pattern resulted in the inclusion of a substantial number of patients with severe or decompensated cirrhosis, including hospitalized patients with acute decompensation. To address the resulting heterogeneity in disease severity, analyses were stratified according to Child-Pugh class. Exclusion criteria were chronic kidney disease, acute-on-chronic liver failure, history of portal vein thrombosis, venous thromboembolism, intra- or extrahepatic solid tumor, e.g., hepatocellular carcinoma, or hematologic disease, HIV infection, history of organ transplantation, use of anticoagulant, antiplatelet or antifibrinolytic therapy, major surgery less than 1 month before inclusion, blood product transfusion less than 1 week before inclusion, admission to intensive care unit, and transfer from another hospital. Enrolled in-patients were admitted to the hospital with acutely decompensated cirrhosis, ascites, hepatic encephalopathy, or variceal hemorrhage. Included outpatients were patients with a history of decompensation (currently clinically stable) or compensated cirrhosis enrolled at associated liver clinics. All patients were prospectively followed up for a median of 499 days (95% CI: 478–524 days), during which clinical events including (procedure-related) bleeding and thrombotic events were documented. Thirty-four patients had a co-infection with hepatitis B or C virus.

Healthy subjects were recruited at outpatient clinics. Exclusion criteria were history of acute and/or chronic disease, and use of anticoagulants, antibiotics, and/or hormonal therapy. The study was performed in accordance with the Declaration of Helsinki and patients and controls gave written informed consent (HIC #approval of Padua University Hospital Ethics Committee #0034435).

### Sample collection and storage

Blood was collected via peripheral venipuncture from an antecubital vein with a 21-gauge needle into vacuum tubes (1 volume of trisodium citrate 0.109 M to 9 volumes of blood; VACUETTE®, Greiner Bio-One, Kremsmünster, Austria). Plasma samples were processed according to a standardized protocol using double centrifugation and stored at −80 °C until analysis: Platelet poor plasma (PPP) was prepared by centrifugating twice at 2,500 • g for 15 min. PPP aliquots of 0.5 mL were frozen at – 80 °C until analysis. Coagulation testing was performed in November 2023, resulting in a storage duration ranging from approximately 6–18 months.

### Coagulation factors

Antithrombin, prothrombin, and fibrinogen levels were measured on the automated coagulation analyzer STA-R max, according to manufacturer’s specifications (Diagnostica Stago, Asnière-sur-Seine, France). Plasma α_2_Macroglobulin (α_2_M) levels were measured with an in-house chromogenic assay as previously described by Kremers et al. (Kremers et al., 2015a). FVIII activity was measured using factor-deficient plasmas and Dade Actin FS activated PTT Reagent (Siemens Healthcare Diagnostics, United States of America).

### Thrombin generation

Thrombin generation was measured in PPP using the CAT assay (Diagnostica Stago, Asnière-sur-Seine, France), as described in more detail previously (van der Heijden et al., 2021). Thrombin generation was measured using the most sensitive CAT reagent, PPP Reagent Low, in the presence and absence of human recombinant thrombomodulin (in house). TG parameters lag time, time-to-peak, peak, ETP and velocity index were calculated using the dedicated Thrombinoscope software (Version 5.0, Diagnostica Stago, Asnière-sur-Seine, France). The percentage inhibition by TM was calculated as stated in Equations 1, 2, respectively for peak height, and ETP.

$$\left(1-\frac{Peak\;height\;in\;presence\;of\;TM}{Peak\;height\;in\;absence\;of\;TM}\right)*100\%$$ (1)

$$\left(1-\frac{ETP\;in\;presence\;of\;TM}{ETP\;in\;absence\;of\;TM}\right)*100\%$$ (2)

The generated TG curves were used in thrombin dynamics analysis, as described below.

### Thrombin dynamics

The TG curve is the net result of prothrombin conversion and thrombin inactivation. Therefore, the course of prothrombin conversion can be calculated from a TG curve (Kremers et al., 2015a; de Laat-Kremers et al., 2020). The prothrombin conversion curve is quantified by the area under the curve, which is defined as the total amount of prothrombin converted (PC_tot_) during the TG test, and the peak height of the prothrombin conversion curve, which is defined as the maximum rate of prothrombin conversion (PC_max_). In addition, the amount of thrombin-antithrombin (T-AT) and thrombin-α_2_M (T-α_2_M) complexes are quantified during the experiment (Kremers et al., 2015b; de Laat-Kremers et al., 2020; Kremers et al., 2018). The thrombin decay capacity (TDC) quantifies the rate of thrombin inactivation as is the pseudo-first order decay constant for thrombin inhibition by antithrombin, α_2_M and fibrinogen (Kremers et al., 2015a).

### Statistics

Statistical analyses were performed in GraphPad Prism (version 10.2.1, GraphPad Software, Boston, Massachusetts United States of America) and SPSS (version 31, IBM Corp., Armonk, NY, United States of America). Normality of the data was analyzed using the Shapiro-Wilk test. Differences between patients and controls were assessed using the T-test, Mann Whitney test, ANOVA analysis or Kruskal–Wallis testing, according to the distribution of the data and the number of groups in the comparison. Post hoc corrections were used in combination with ANOVA (Bonferroni) and Kruskal–Wallis testing (Dunn). Heat map analysis was performed to visualize the footprint of mean values of thrombin dynamics parameters in patients without hemostatic events, and patients with bleeding or thrombotic episodes. Cox proportional hazards regression was used to assess the association between individual thrombin dynamics parameters and the occurrence of bleeding or thrombosis during follow-up. Models were run both unadjusted and adjusted for age and sex. Patients were also dichotomized according to the lowest quintile of the relevant parameter, and hazard ratios were calculated for these groups, again with and without adjustment for age and sex. P-values below 0.05 were considered statistically significant.

## Results

Thrombin generation and coagulation factor levels were measured in 101 liver cirrhosis patients and 20 controls (Tables 1 and 2). Patients were grouped according to the Child-Pugh score (Table 1). Thirty-two patients (33.7%) were scored as CP-A, 22 (23.2%) as CP-B, and 41 (43.3%) as CP-C. Patients were older than controls (63 vs. 35 years of age on average, p < 0.0001). Cirrhosis patients were more often male (70%) than female (30%). Cirrhosis was attributed to alcohol use in 52% of patients, and to viral and metabolic causes in 34% and 14% of patients, respectively. Four bleeding and 7 thrombotic events were recorded during the median follow-up period of 499 days.

**TABLE 1:** Baseline characteristics.

Parameter	Controls	All patients	CP-A	CP-B	CP-C
N	20	101	32	22	41
Age	35 ± 14	63 ± 10****	67 ± 10	63 ± 10	59 ± 10**
Sex (% male)	50%	70%	75%	77%	62%
Etiology					
Alcohol, n (%)	-	53 (52%)	15 (47%)	8 (36%)	28 (72%)
Viral, n (%)	-	34 (34%)	14 (44%)	8 (36%)	8 (20%)
Metabolic, n (%)	-	10 (14%)	2 (6%)	4 (18%)	4 (10%)
Other, n (%)	-	4 (6%)	1 (3%)	2 (9%)	1 (3%)
Reasons for admission					
Ascites, n (%)	-	-	-	-	27 (66%)
Hepatic encephalopathy, n (%)	-	-	-	-	8 (20%)
Variceal hemorrhage (%)	-	-	-	-	5 (12%)
Acute kidney injury, n (%)	-	-	-	-	1 (2%)
Laboratory parameters					
MELD score	-	16 ± 7	14 ± 16	14 ± 5	23 ± 6
Platelet count (10^9^/L)	-	88 ± 57	109 ± 60	82 ± 77^++^	75 ± 31^+^
C-reactive protein (mg/L)	-	12.8 ± 16.1	3.8 ± 5.4	17.1 ± 21.2^+++^	18.2 ± 15.9^++++^

Abbreviations: CP: Child-Pugh, MELD: Model End Stage Liver Disease. **p < 0.01, ****p < 0.0001 comparing patients to controls. ^+^p < 0.05, ^++^p < 0.01, ^+++^p < 0.001, ^++++^p < 0.0001 comparing CP-B, and CP-C, to CP-A, patients.

**TABLE 2:** Hemostatic variables.

Test parameter	Controls	All patients	P-value	CP-A	CP-B	CP-C	P-value
Thrombin generation							
Lag time (min)	4.2 ± −0.4	4.2 ± 1.4	n.s	4.3 ± 0.8	4.0 ± 1.0	4.1 ± 1.3	n.s
Peak height (nM)	112 ± 50	134 ± 48	0.039	159 ± 47	141 ± 39	113 ± 42	<0.0001
Time-to-peak (min)	9.1 ± 1.4	7.5 ± 2.0	<0.0001	7.8 ± 13	7.2 ± 1.7	7.2 ± 1.8	n.s
ETP (nM•min)	934 ± 204	868 ± 223	n.s	999 ± 196	909 ± 182	750 ± 205	<0.0001
Velocity index (nM/min)	26 ± 18	44 ± 20	0.0002	49 ± 20	48 ± 21	39 ± 18	0.0462
Effect of thrombomodulin							
Peak height inhibition (%)	19 ± 8	4 ± 5	<0.0001	5 ± 7	5 ± 4	3 ± 4	0.0367
ETP inhibition (%)	45 ± 14	13 ± 12	<0.0001	18 ± 12	13 ± 9	8 ± 9	<0.0001
Coagulation factor levels							
Fibrinogen (g/L)	2.6 ± 0.7	2.5 ± 1.2	n.s	3.4 ± 0.9	2.4 ± 1.0	1.9 ± 1.1	<0.0001
Prothrombin (%)	86 ± 8	46 ± 19	<0.0001	62 ± 12	45 ± 11	33 ± 15	<0.0001
FVIII (%)	118 ± 12	203 ± 77	<0.0001	176 ± 45	202 ± 66	228 ± 93	0.0385
Antithrombin (%)	99 ± 13	55 ± 26	<0.0001	75 ± 18	50 ± 18	39 ± 22	<0.0001
α2-Macroglobulin (μM)	3.8 ± 1.3	5.5 ± 2.6	0.0009	7.8 ± 2.5	5.0 ± 2.3	4.2 ± 1.6	<0.0001

Abbreviations: CP: Child-Pugh, ETP: endogenous thrombin potential, FVIII: Factor VIII.

Quantification of thrombin generation parameters and coagulation factor levels.

TG was measured using PPP Reagent Low (Table 2). TG peak height and velocity index were significantly higher in patients compared to controls (134 ± 48 nM vs. 112 ± 50 nM, p = 0.039; and 44 ± 22 nM/min vs. 26 ± 18 nM/min, p = 0.0002; respectively). Time-to-peak was shorter in patients (7.5 ± 2.0 min vs. 9.1 ± 1.4 min, p < 0.0001, Table 2). Peak height, velocity index and ETP decreased significantly with the severity of the disease (CP-A vs. CP-B vs. CP-C; Table 2). Peak height significantly decreased form 159 ± 47 nM in Cp-A patients to 141 ± 39 nM in CP-B patients, to 113 ± 42 in CP-C patients (p < 0.0001). Velocity index decreased significantly from 49 ± 20 nM/min to 48 ± 21 nM/min to 39 ± 18 nM/min, respectively in CP-A, CP-B and CP-C patients (p = 0.0462). Moreover, ETP was 999 ± 196 nM⋅min in CP-A patients, compared to 909 ± 182 in CP-B patients, and 750 ± 205 nM in CP-C patients (p < 0.0001). The addition of thrombomodulin to the TG assay revealed a significant APC pathway desensitization in cirrhosis patients (13% vs. 45% inhibition of the ETP, p < 0.0001), which increased with cirrhosis severity (18% vs. 13% vs. 8% in CP-A. CP-B, and CP-C patients, respectively; p < 0.0001). Moreover, plasma fibrinogen, prothrombin and antithrombin decreased significantly with disease severity, and prothrombin (46% ± 19% vs. 86% ± 8%, p < 0.0001) and antithrombin levels (55% ± 26% vs. 99% ± 13%, p < 0.0001) were significantly lower in patients than controls (Table 2). On the contrary, FVIII (203% ± 77% vs. 118% ± 12%, p < 0.0001) and α_2_-Macroglobulin levels (5.5 ± 2.6 μM vs. 3.8 ± 1.3 μM, p = 0.0009) were significantly higher in patients than controls (Table 2).

**FIGURE 1:**
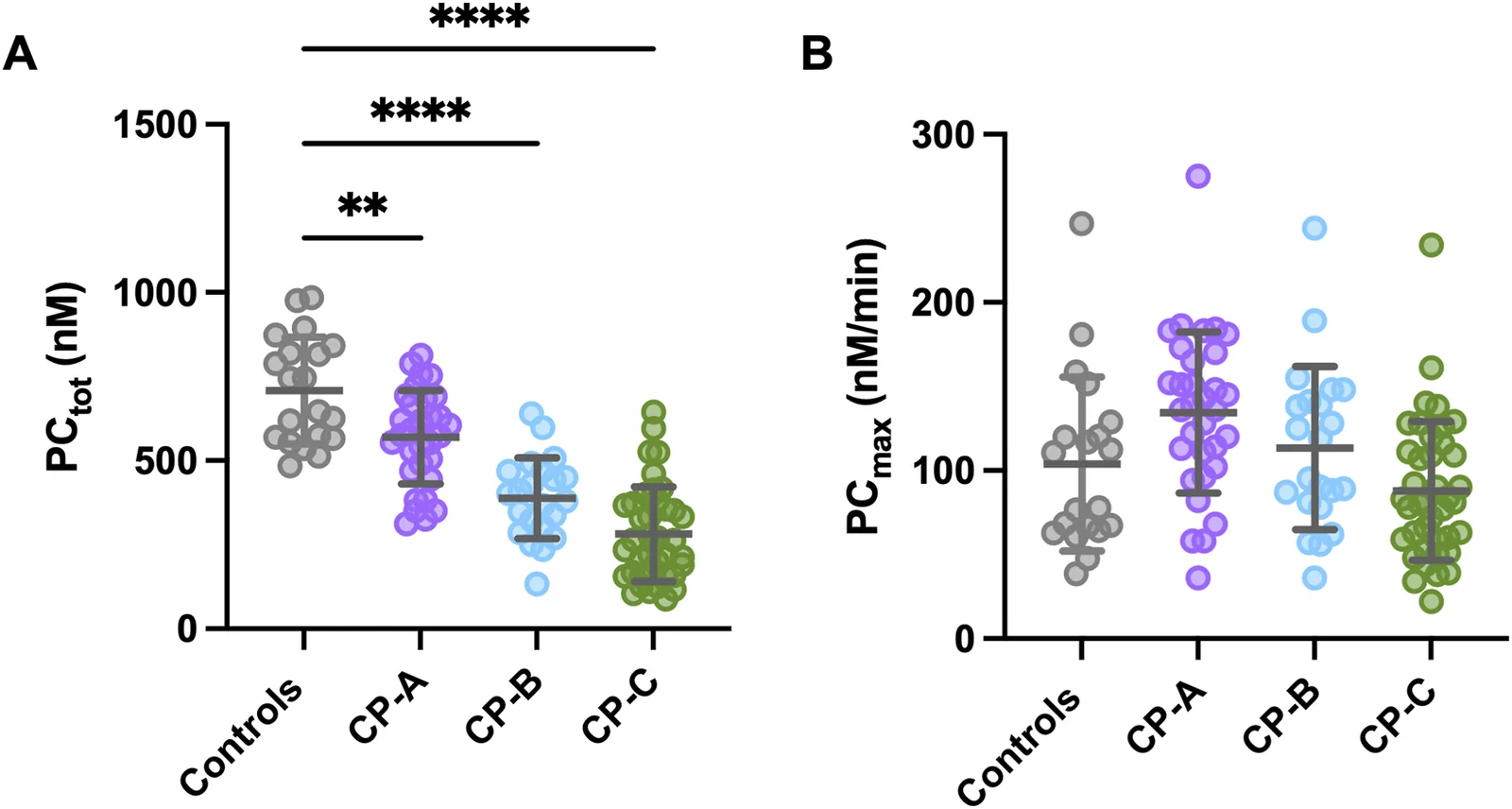
Prothrombin conversion in cirrhosis patients and controls. **(A)** The total amount of prothrombin converted during thrombin generation. **(B)** The maximum rate of prothrombin conversion during thrombin generation. **p < 0.01, ****p < 0.0001.

We applied thrombin dynamics analysis to TG data to investigate the balance of TG by studying the alterations in prothrombin conversion and thrombin inactivation in liver cirrhosis. The amount of prothrombin converted (PC_tot_) during TG was significantly reduced in patients compared to controls (−45%, p < 0.0001; Figure 1A), and the magnitude of PC_tot_ reduction was associated with the CP score. In contrast, the rate of prothrombin conversion (PC_max_) was unaltered in cirrhosis patients (Figure 1B). Both PC_tot_ and PC_max_ were significantly correlated with the plasma prothrombin level, as expected, with respective Pearson’s correlation coefficients of 0.87 (p < 0.001) and 0.30 (p = 0.001).

**FIGURE 2:**
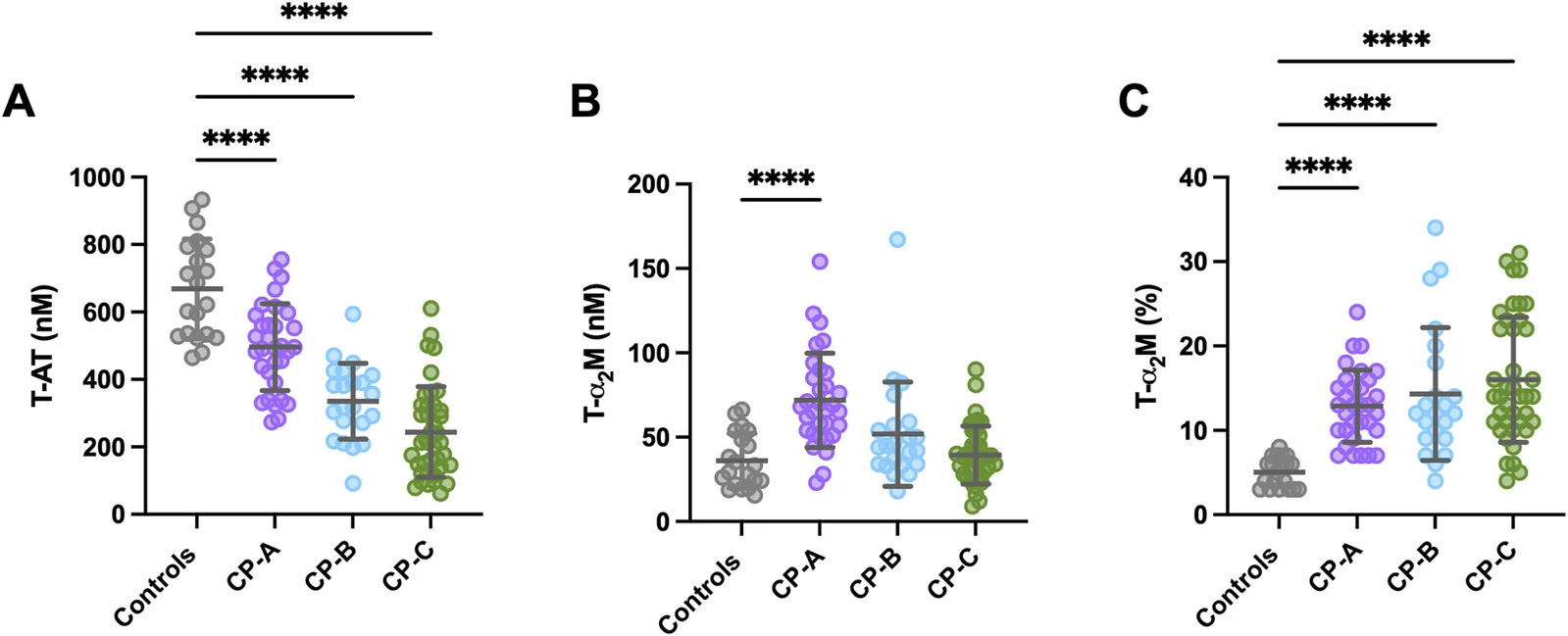
Thrombin inactivation in cirrhosis patients and controls. **(A)** Thrombin-antithrombin complex formation during thrombin generation. **(B)** Thrombin-α2-macroglobulin complex formation during thrombin generation. **(C)** The relative contribution of α2-macroglobulin to the inhibition of thrombin during thrombin generation. ****p < 0.0001.

The inactivation of thrombin during TG was quantified as the formation of thrombin-antithrombin (T-AT) and thrombin-α_2_-Macroglobulin (T-α_2_M) complexes (Figure 2). The formation of T-AT was significantly lower in CP-A (−26%, p < 0.0001), CP-B (−50%, p < 0.0001) and CP-C (−64%, p < 0.0001) cirrhosis patients compared to controls (Figure 2A). The formation of T-α_2_M complexes was higher in CP-A patients than controls (+48%, p = 0.0064; Figure 2B). Moreover, the percentage of thrombin inhibition by α_2_M was significantly higher in CP-A, CP-B and CP-C cirrhosis patients compared to controls (+136%, p < 0.0001); Figure 2C). T-AT formation was significantly correlated with the plasma prothrombin level (Pearson’s R = 0.89, p < 0.001), antithrombin level (Pearson’s R = 0.92, p < 0.001), and α_2_M level (Pearson’s R = 0.19, p = 0.040). In contrast, T-α_2_M formation was not significantly correlated with either prothrombin, antithrombin or α_2_M levels.

**FIGURE 3:**
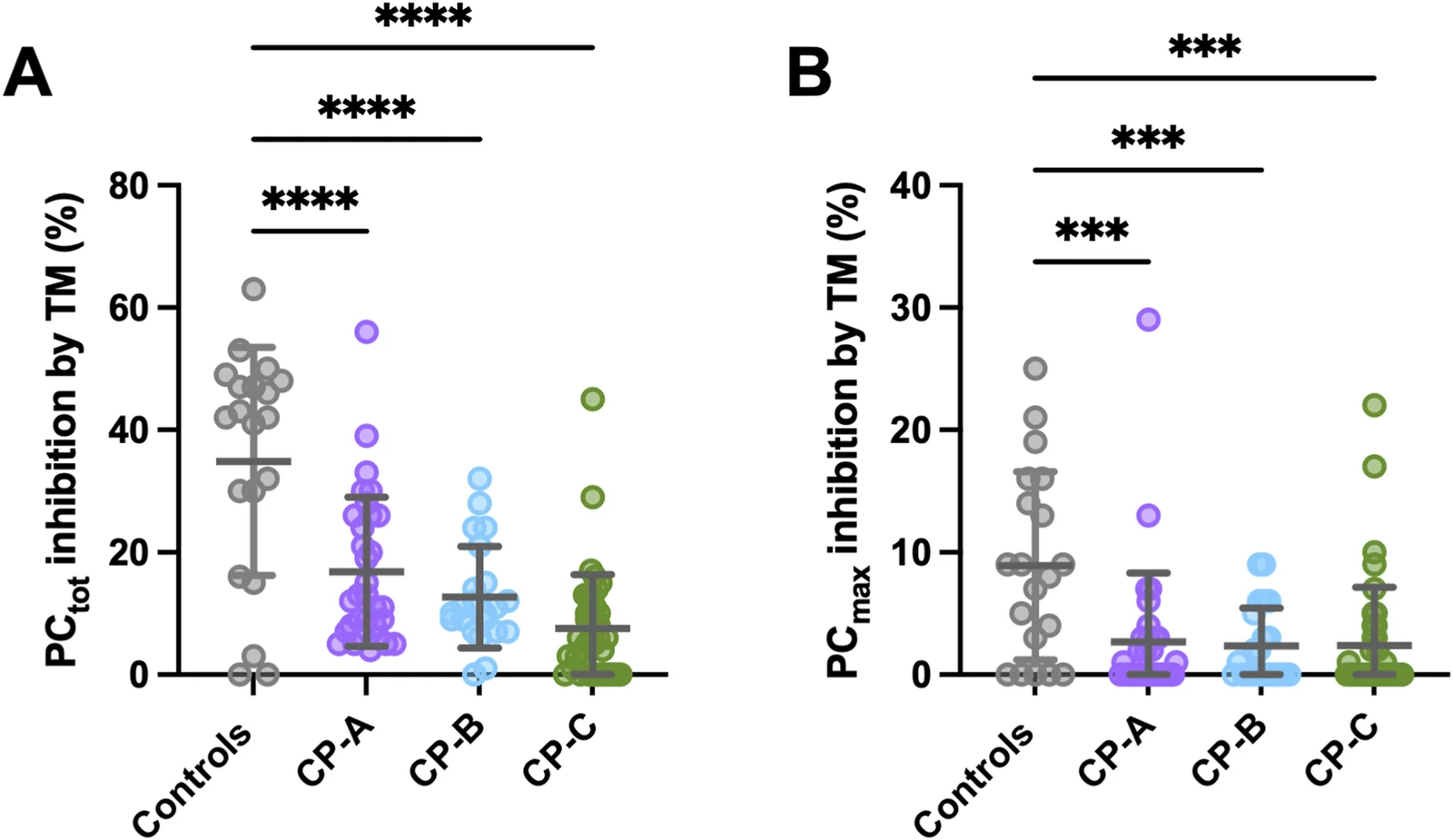
Thrombomodulin sensitivity in cirrhosis patients and controls. **(A)** Inhibition of the total amount of prothrombin converted during thrombin generation in the presence of thrombomodulin. **(B)** Inhibition of the maximum rate of prothrombin conversion during thrombin generation in the presence of thrombomodulin. ***p < 0.001, ****p < 0.0001.

As for TG, the inhibitory actions of TM are much less pronounced in cirrhosis patients compared to controls. Both the total amount of prothrombin conversion (9.0% vs. 42%, p < 0.0001), as well as maximum rate of prothrombin conversion (0.0% vs. 8.5%, p < 0.0001) are significantly less inhibited by TM in cirrhosis patients than controls (Figure 3).

**FIGURE 4:**
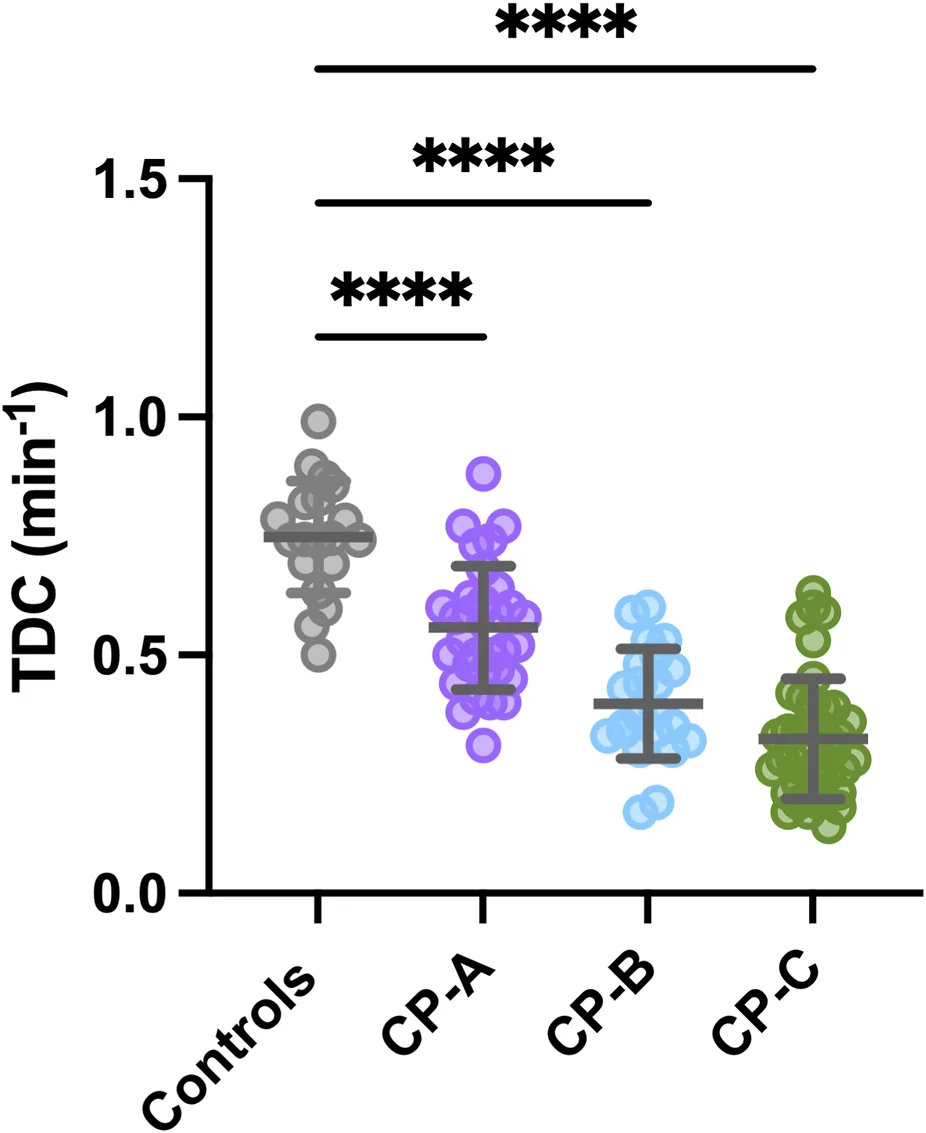
The thrombin decay rate in cirrhosis patients and controls. The thrombin decay capacity was determined independently from the thrombin generation curve, based on the plasma levels of antithrombin, α2-macroglobulin and fibrinogen. ****p < 0.0001.

Independent of TG, the thrombin decay capacity was determined to quantify the thrombin inhibitory potential of each subject (Figure 4). Cirrhosis patients had significantly less thrombin inhibitory potential than controls (0.410 min^-1^ vs. 0.743 min^-1^, p < 0.0001) and the reduction of TDC was associated with disease severity (0.558 min^-1^ vs. 0.398 min^-1^ vs. 0.324 min^-1^ for CP-A, CP-B, CP-C, respectively; p < 0.0001).

**FIGURE 5:**
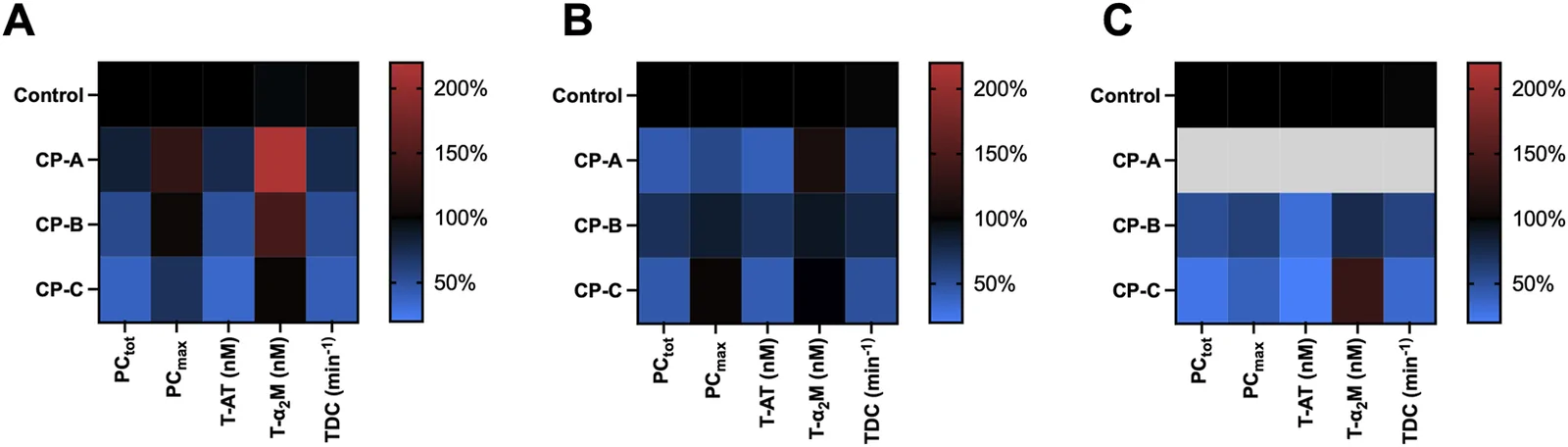
Relative thrombin dynamics parameter values in cirrhosis patients compared to controls. **(A)** Heat map of thrombin dynamics parameter values stratified for Child-Pugh status in event-free cirrhosis patients. **(B)** Heat map of thrombin dynamics parameter values stratified for Child-Pugh status in patients with thrombosis during follow-up. **(C)** Heat map of thrombin dynamics parameter values stratified for Child-Pugh status in patients with bleeding during follow-up.

We used heat map analysis to determine a cirrhosis-specific footprint of thrombin dynamics parameter (Figure 5). Compared to controls, PC_tot_, T-AT and TDC were increasingly reduced with increasing disease severity (Figure 5A, p < 0.001). Conversely, T-α_2_M formation was increased in CP-A and CP-B patients (p < 0.0001). We performed separate heat map analyses for patients that had reported thrombotic (Figure 5B, n = 7) or bleeding complications (Figure 5C, n = 4). Four thrombotic events were recorded as portal vein thrombosis, and 3 deep venous thrombosis. All four bleeding events were major, procedure-related bleeding events: two post-paracentesis hemoperitoneum events, one bleeding event following dental extraction, and one bleeding event following sphincterotomy during endoscopic retrograde cholangiopancreatography (ERCP). The median time from inclusion to the bleeding event was 16 days (IQR 7–35 days). Patients that had experienced thrombosis tended to have lower PC_tot_, T-AT and TDC values, especially CP-A patients (Figure 5B). Bleeding only occurred in CP-B and CP-C patients. Patients that suffered from procedure-related bleeding problems had low PC_max_ values (p = 0.0292) in addition to a trend towards lower PC_tot_, T-AT and TDC, and higher T-α_2_M (Figure 5C).

**FIGURE 6:**
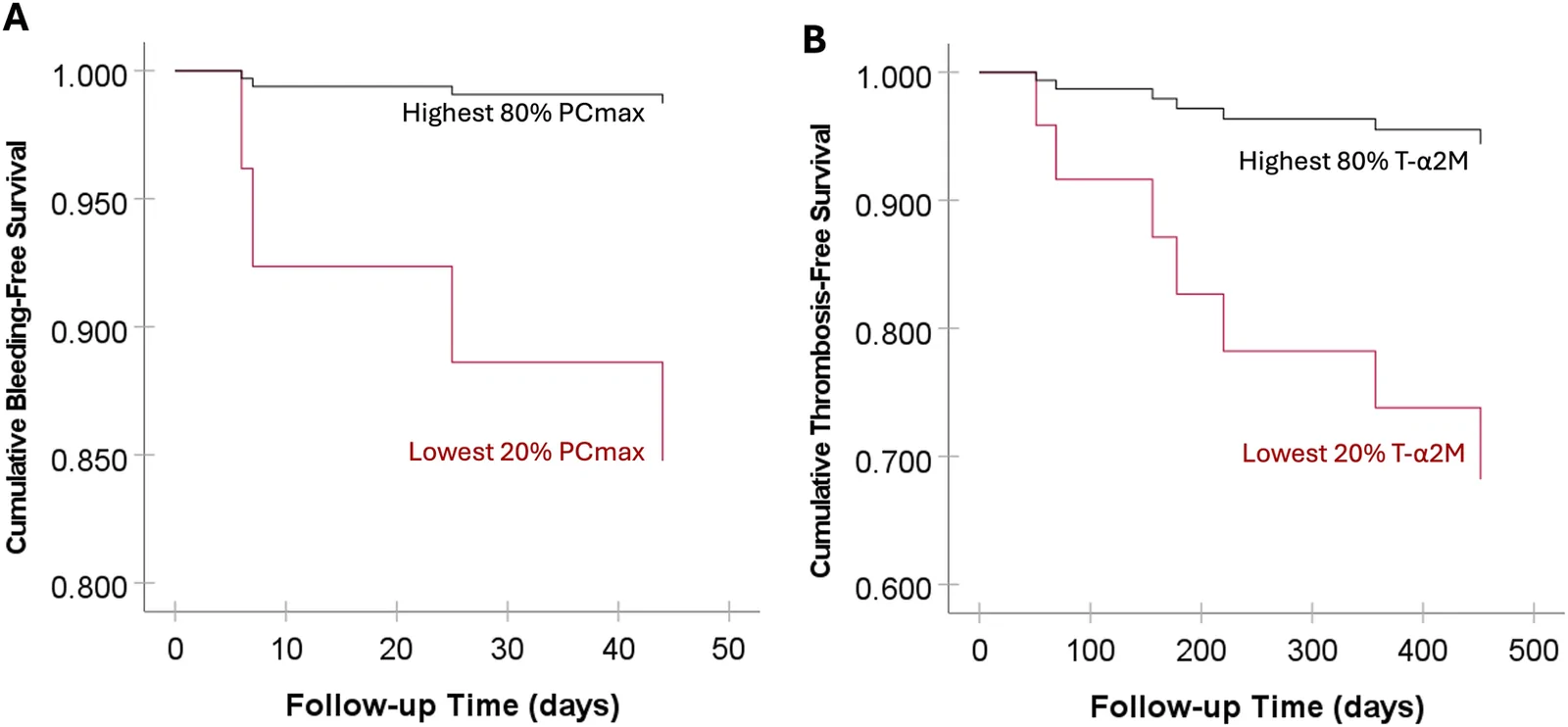
Bleeding-free and thrombosis-free survival stratified by thrombin dynamics parameters. **(A)** Survival curves for bleeding-free survival in subjects in the lowest PCmax quintile compared to the remainder of the patients. **(B)** Survival curves for thrombosis-free survival in subjects in the lowest T-α2M quintile compared to the remainder of the patients.

We further investigated the association of thrombin dynamics parameters with bleeding and thrombotic episodes using survival analysis. Table 3 summarizes the hazard ratios for each thrombin dynamics parameter for bleeding and thrombosis obtained using Cox regression analyses on the crude thrombin dynamics parameters. Thrombosis during follow-up was significantly associated with lower T-a2M formation, whereas low PC_max_ rates were associated with an increased hazard of bleeding. Adjustment of the regression models for age and sex did not significantly alter the results (data not shown). The association of low PC_max_ values and bleeding was further explored by dividing the cohort into subjects with the lowest 20 percent of PC_max_ values, and the remaining 80% with higher PC_max_ levels (Figure 6A). Patients in the lowest PC_max_ category had a HR of 12.9 (95% CI 1.34–124.31, p = 0.027) for developing bleeding. Adjustment for age and sex did not significantly affect the association, resulting in a HR of 12.6 (95% CI 1.22–129.83, p = 0.034). Similarly, the association of low T-α_2_M levels and thrombosis were explored, revealing that patients in the lowest quintile of T-α_2_M levels had a HR of 6.6 (95% CI 1.47–29.9, p = 0.014), for the development of thrombosis (Figure 6B). Adjustment for age and sex strengthened the association, resulting in a HR of 7.35 (95% CI 1.46–36.87, p = 0.015).

**TABLE 3:** Cox regression analysis for the association of thrombin dynamics parameters and bleeding and thrombotic episodes.

Thrombosis			Bleeding		
N of events (rate)	7 (6.9)	p-value	N of events (rate)	4 (3.9)	p-value
PC_tot_ (nM)	0.997 (0.993–1.002)	0.229	PC_tot_ (nM)	0.995 (0.989–1.002)	0.166
PC_max_ (nM/min)	0.992 (0.975–1.009)	0.343	PC_max_ (nM/min)	0.953 (0.913–0.994)	0.026
T-AT (nM)	0.998 (0.993–1.002)	0.330	T-AT (nM)	0.995 (0.988–1.002)	0.164
T-α_2_M (nM)	0.956 (0.914–1.000)	0.049	T-α_2_M (nM)	0.975 (0.927–1.027)	0.344
TDC (min^-1^)	0.462 (0.004–58.243)	0.754	TDC (min^-1^)	0.171 (0.000–99.67)	0.587

## Discussion

We investigated the association of cirrhosis-induced changes in prothrombin conversion and thrombin inactivation with bleeding and thrombosis episodes. The total amount of prothrombin converted (PC_tot_) during TG is significantly reduced in liver cirrhosis patients, which is most likely due to lower prothrombin levels (Tripodi et al., 2011b; Kremers et al., 2015a). Furthermore, low PC_max_ was shown to be significantly associated with a higher hazard of bleeding episodes in cirrhosis patients. In addition, low thrombin-α_2_M formation was significantly associated with an increased risk of thrombosis.

Thrombin dynamics results confirm our earlier findings of a reduction of PC_tot_ in a case-control cohort of liver cirrhosis patients, for which, unfortunately, no follow-up data on bleeding and thrombosis was available (Kremers et al., 2017). The formation of thrombin-inhibitor complexes during TG heavily relies on the amount of thrombin formed (Kremers et al., 2015a), and therefore T-AT and T-α2M formation were reduced in cirrhosis patients. Our study confirms the reports of reduced AT and prothrombin levels (Senzolo et al., 2012; Potze et al., 2013; Delahousse et al., 2010; Tripodi et al., 2013; Kremers et al., 2017; Gatt et al., 2010) and increased α2M and fibrinogen levels (Northup et al., 2006; Lee et al., 2009; Imbert-Bismut et al., 2001; Ho et al., 2010; Gangadharan et al., 2007) in cirrhosis patients. Moreover, thrombin decay capacity was reduced in cirrhosis patients, relative to disease severity. Even though α2M levels are increased in cirrhosis, α2M is incapable of restoring the lower thrombin decay capacity caused by low AT levels. Intriguingly, the increase of fibrinogen levels reduced the thrombin inhibitory potential even further in CP-A patients compared to controls, as fibrinogen competes with AT and α2M for thrombin binding, preventing thrombin from being inhibited (Kremers et al., 2015a).

The net effect of the reduction of prothrombin conversion and the attenuation of thrombin inactivation, was an increase of TG peak height, whilst ETP remained unchanged, as reported in the past (Lisman et al., 2022; Northup et al., 2006; Tripodi et al., 2005; Tripodi et al., 2011b; Lisman et al., 2010; Kremers et al., 2017; Gatt et al., 2010; Youngwon et al., 2013; Chaireti et al., 2014; Groeneveld et al., 2014). Notably, the sensitivity of thrombin generation assays to anticoagulant deficiencies depends strongly on the tissue factor (TF) concentration used to trigger the reaction. Assays performed at low TF concentrations, such as the PPP-Reagent Low reagent used in this study, are considerably more sensitive to reductions in protein C, protein S and antithrombin than assays triggered with intermediate or high TF concentrations, in which the procoagulant drive can mask underlying anticoagulant deficiencies (Lisman et al., 2022; Tripodi et al., 2005; Lisman et al., 2021; Winckers et al., 2025). This may explain why the reduced anticoagulant capacity observed in our cohort—reflected by the lower thrombin decay capacity—remained clearly detectable despite a relatively preserved or even increased thrombin generating potential.

TG triggered using PPP Reagent Low is relatively more dependent on amplification via the intrinsic tenase complex, in which both FVIII and FXI (through thrombin-mediated feedback activation) contribute more than under higher-TF conditions. FVIII levels were markedly elevated in our cohort (Table 2), consistent with a more procoagulant intrinsic pathway contribution; FXI levels were not measured in this study, which limits our ability to quantify its contribution and should be addressed in future work.

Even though a new hemostatic equilibrium seems to be established in liver cirrhosis, patients are at risk for bleeding and thrombosis [ (Lisman and Porte, 2010; Northup et al., 2006; Sharara and Rockey, 2001; Northup et al., 2008; Mor et al., 1993; Ambrosino et al., 2017). In part this may be explained by non-hemostatic mechanistic causes such as portal hypertension, but a loss of hemostatic buffer capacity has been proposed as a cause for the elevated hemostatic risk in cirrhosis patients (Kremers et al., 2017; Zanet et al., 2023). These findings support the concept of rebalanced hemostasis in cirrhosis, in which concomitant changes in procoagulant and anticoagulant pathways result in a fragile new equilibrium rather than a simple bleeding phenotype. Reduced prothrombin conversion was counterbalanced by attenuated thrombin inactivation, helping preserve thrombin generation despite marked factor changes. The associations of low PCmax with bleeding and low T-α2M with thrombosis suggest that this state is fragile and prone to shift in either direction. Thrombin dynamics may therefore provide a more mechanistic view of hemostatic balance than conventional coagulation tests. Similar shifts have also been described in children and during cardiopulmonary bypass (Kleinegris et al., 2014; Kremers et al., 2016).

We investigated the association between prothrombin conversion and thrombin inactivation parameters with clinically relevant bleeding and thrombotic episodes. In heat map analyses, we found two distinctly different thrombin dynamics profiles in patients that developed procedure related bleeding events and portal vein and extra-splanchnic thrombosis during follow-up. Moreover, patients that did not suffer from bleeding or thrombotic episodes had higher T-α_2_M levels than patients that did develop hemostatic events. Additionally, subjects in the lowest quintile for T-α_2_M were significantly at risk for thrombosis. This indicates that T-α_2_M could not only be seen as a potential indicator of bleeding or thrombotic risk, but also as a marker of stability of the hemostatic balance. Indeed, high levels of T-α_2_M would suggest that α_2_M has taken over the function of AT to a large enough extent to allow a more stable hemostatic balance, even though coagulation factor levels overall are reduced. Conversely, reduced T-α_2_M levels indicate a state in which a smaller proportion of generated thrombin is neutralized by endogenous inhibitors, reflecting both already diminished antithrombin activity and insufficient compensatory engagement of α_2_M. As a result, a greater amount of free, enzymatically active thrombin remains available in the circulation, which may promote downstream procoagulant processes such as fibrin polymerization and platelet activation. This provides a biologically plausible mechanistic link between low T-α_2_M levels and an increased risk of thrombosis, although this interpretation should be regarded as a mechanistic hypothesis requiring further experimental validation rather than a definitive causal explanation (Zanetto et al., 2025). Although the study was underpowered to make a statistically significant distinction between patients suffering from bleeding and thrombotic episodes, we found that patients at risk for bleeding had a significantly lower maximum prothrombin conversion rate. These data therefore provide valid exploratory evidence that thrombin dynamics parameters, particularly PC_max_ and T-α2 M, may be linked to bleeding and thrombotic events in cirrhosis. However, given the limited power of the study and the exploratory nature of part of the analyses, these findings should be interpreted as hypothesis-generating rather than predictive or mechanistically definitive. Other limitations of the study are the number of bleeding and thrombotic event, the lack of non-procedure related bleeding events, the age difference between patients and controls, and the heterogenous nature of the patient group due to the enrollment of CP-A, CP-B, and CP-C patients (Zanetto et al., 2025). Nevertheless, the latter allowed us to investigate the changes in thrombin dynamics in association with the severity if the disease. The prognostic data in this study should be confirmed in a larger prospective cohort study, including follow-up data for non-procedure related bleeding events. Additionally, samples were not collected on corn trypsin inhibitor, and therefore, a minor contribution of contact-pathway (FXII-driven) medicated activation of thrombin generation cannot be ruled out. Likewise, the study did not examine FXI levels, so their impact on our results cannot be evaluated here.

In conclusion, our findings show that reduced prothrombin conversion and thrombin inactivation contribute to a rebalanced thrombin generation profile in patients with cirrhosis. We also observed distinct differences in thrombin dynamics parameters that may be associated with either bleeding or thrombosis in this population. These observations require confirmation in a large, dedicated cohort to better define the value of thrombin dynamics parameters in assessing hemostatic imbalance in cirrhosis.

## Data Availability

The original contributions presented in the study are included in the article/supplementary material, further inquiries can be directed to the corresponding author.
